# Safe dose of intravitreal imatinib and its effect on laser-induced choroidal neovascularization: a rat-model experiment

**DOI:** 10.1186/s40942-015-0017-4

**Published:** 2015-10-01

**Authors:** Homayoun Nikkhah, Hamid Ahmadieh, Alireza Ramezani, Mozhgan Rezaei Kanavi, Seyed Bagher Hosseini, Naficeh Sadeghi, Seyed Mohsen Khandaghy Meybodi, Mehdi Yaseri

**Affiliations:** 1 Ocular Tissue Engineering Research Center, Shahid Beheshti University of Medical Sciences, Tehran, Iran; 2 Ophthalmic Research Center, Shahid Beheshti University of Medical Sciences, Tehran, Iran; 3 Ophthalmic Epidemiology Research Center, Shahid Beheshti University of Medical Sciences, Tehran, Iran; 4 Department of Drug and Food Control, School of Pharmacy, Tehran University of Medical Sciences, Tehran, Iran; 5 Department of Biostatistics and Epidemiology, Tehran University of Medical Sciences, Tehran, Iran; 6 Ophthalmic Research Center, No. 23, Boostan 9 St., Pasdaran Ave, Tehran, 16666 Iran

**Keywords:** Imatinib, Choroidal neovascularization, Platelet derived growth factor, Rat, Fluorescein angiography

## Abstract

**Background:**

This two-phase experimental study was conducted to determine the maximum safe dose of intravitreal imatinib (IVI) and its inhibitory effect on a rat model of choroidal neovascularization (CNV).

**Methods:**

In phase I, 60 rats were divided into six groups (A to F); five of which received IVI with concentrations of 330 (A), 250 (B), 165 (C), 80 (D), and 40 (E) µg/5 µl, and the control group (F) received balanced salt solution (BSS). In addition to electroretinography (ERG), routine histopathological analysis and immunohistochemistry for glial fibrillary acidic protein were performed. In phase II, CNV was induced by laser photocoagulation in 25 rats and the animals were divided into two groups. One group received the maximum safe dose of IVI, determined in phase I, and the other received intravitreal BSS. After 4 weeks, the groups were compared in terms of mean scores of fluorescein leakage in fluorescein angiography and the mean CNV areas in histopathological sections.

**Results:**

In phase I, ERG and the histopathological findings revealed retinal toxicity in groups A to D and A to C, respectively; therefore, a dose of 40 µg/5 µl imatinib was specified as the maximum safe dose for phase II. In phase II, late phase fluorescein leakage and the CNV areas were not significantly different between the imatinib-treated eyes and the controls (p = 0.62 and p = 0.5, respectively).

**Conclusions:**

Despite the safety of IVI with a dose of 40 µg/5 µl, no inhibitory effect on laser-induced CNV was observed. Further studies are required to investigate the possible synergistic effects of Imatinib with conventional anti-CNV drugs.

## Background

Age-related macular degeneration (AMD) is the leading cause of legal blindness in patients aged over 65 [1] and the most common cause of blindness in the Western world [2]. Approximately 10 % of AMD patients manifest with the neovascular form of the disease [2]. It has been shown that the vascular endothelial growth factor (VEGF) is the most important cytokine in both physiological and pathological angiogenesis, which leads to the formation of choroidal neovascularization (CNV) [3–5]. Therefore, nowadays anti-VEGF agents are the mainstay of anti-angiogenesis therapy.

Platelet-derived growth factors (PDGF) as the second important cytokines involved in angiogenesis [6], facilitate the recruitment of pericytes and smooth muscle cells and are essential for maturation and stability of the vasculature [7, 8]. The PDGF family binds to two different receptors, known as the PDGF receptor alpha and beta. The PDGF receptors (PDGFR) are tyrosine kinases, with PDGFR-β being more extensively expressed in endothelial cells and vascular smooth muscle cells [9–11]. Tyrosine kinases are important cellular signaling proteins. Multiple tyrosine kinases are involved in angiogenesis, including receptor tyrosine kinases such as VEGF and PDGF receptors. Receptor tyrosine kinases are essential for the transduction of extracellular signals into the cell [6]. Several receptor tyrosine kinase inhibitors like sorafenib, cediranib, sunitinib, pazopanib were recently approved [12–15]. These drugs, by inhibition of VEGF and PDGF signaling, may be promising new therapeutic approach to CNV treatment.

Imatinib, a specific inhibitor of tyrosine kinase, was approved in 2001 for the treatment of human cancer [16]. It downregulates PDGFR signal transduction [17]. In the absence of PDGFR signaling, the vascular pericytes strip off and the integrity of the vessel wall is compromised. These vessels become more sensitive to the withdrawal of VEGF, and therefore the vessel regression is more prominent [18–20]. Hence, with this hypothesis that imatinib could inhibit or at least slow down the progression of CNV, the present study was designed in two phases. In phase I, the maximum safe dose of intravitreal imatinib was determined in a rat model, and in phase II, the probable inhibitory effect of intravitreal imatinib on laser-induced CNV was investigated in a rat model.

## Methods

### Study design and animals

This two-phase study was designed to determine the maximum safe dose of intravitreal imatinib (IVI) in phase I and its possible inhibitory effect in an animal model of laser-induced CNV in phase II. Eighty-five Lister Hooded pigmented rats (Razi Institute for Vaccine and Serum Research, Karaj, Iran) weighing 200–300 g were included. The animals were preserved in plastic cages under a 12/12-h dark–light cycle, with access to the food and water ad libitum. The research was ethically reviewed and approved by ethical committee of Ophthalmic Research Center, Shahid Beheshti University of Medical Sciences and adhered to the guidelines of the Association for Research in Vision and Ophthalmology (ARVO) Statement for the use of animals in ophthalmic and vision research and Shahid Beheshti Medical University guidelines on the use of laboratory animals. For all procedures, the animals were anesthetized with intramuscular injection of ketamine hydrochloride (Ketamine-Rotex, Rotexmedica, Trittau, Germany) (80 mg/kg) and xylazine hydrochloride (Xylazin, Animedic, Germany) (5 mg/kg). The pupils were dilated with one drop of tropicamide 0.5 % (Mydrax, Sina Darou, Tehran, Iran). After last ERG and fluorescein angiography in phase I and II respectively, the rats were immediately euthanized by cervical dislocation when they were deeply anesthetized.

### Preparation of intravitreal imatinib

At the present time, imatinib is marketed only in the form of 100 and 400 mg tablets and does not have any injectable form. The active substance of the drug is the mesylate salt form of imatinib, which is a phenylaminopyrimidine derivative in the form of white to slightly yellowish crystalline photostable powder. The solubility of imatinib mesylate in aqueous solutions depends on the pH and its highest solubility is at pH 5.5–5.8. The stock solution of imatinib mesylate (Sigma-Aldrich Chemie GmbH, Taufkirchen, Germany) was prepared in sterile distilled water wherein a pH of 5.5–5.8 was reached using a tiny amount of sulfuric acid. However, after adding imatinib powder, the pH of final solution was about neutral. The amounts of 40, 80, 165, 250 and 330 mg of pure powder were weighted in 5 ml volumetric flasks, dissolved with the distilled water and then made the volume to prepare 40, 80, 165, 250 and 330 µg/5 µl concentrations.

## Phase I

Sixty rats were categorized into six groups (A to F); of which five groups received IVI with concentrations of 330 (A), 250 (B), 165 (C), 80 (D), and 40 (E) µg/5 µl in the right eye, control group (F) received balanced salt solution (BSS). Injections were performed under sterile conditions, using a surgical microscope, by an expert ophthalmologist (HN), who was not aware of the doses, by using a Hamilton 5 µl glass needle just posterior to the limbus. Electroretinography (ERG) examinations were carried out at baseline, and then 1 and 4 weeks after the injection. After 4 weeks, all rats were euthanized and their right eyes were enucleated for histopathological analyses. On the basis of the ERG and light microscopic results, the maximum safe dose of IVI was chosen for phase II.

### Electroretinography

Electroretinograms were obtained at the baseline, just before injections and at 1 and 4 weeks after imatinib injection. All procedures were done in darkness with infra-red illumination and image converters. ERG was carried out as described before [21]. Briefly, after at least 2-h dark adaptation, the animals were anesthetized and their pupils were dilated. During anesthesia body temperature was maintained between 36 and 37 °C. Then the animals were placed in the recording chamber upon a temperature-controlled heating pad. To record the ERG signals, corneal gold-wire electrodes were implemented. The ERG signals were recorded using the RETI-port/scan 21 electrophysiological diagnostic systems (Roland Consult, Brandenburg, Germany). The scotopic and photopic responses were recorded simultaneously in both eyes. ERG changes were considered significant if b-wave amplitudes were reduced by at least 30 % from the baseline value [21]. The ERG results at different time points, were compared in each group and also between the groups.

### Histopathological and immunohistochemical examinations

After the last ERG record, the animals were euthanized and their enucleated eyes fixed in 10 % formalin, were sent for light microscopic examinations. After bisecting each eye axially into two calottes and embedding in paraffin blocks; 5-µm tissue sections were stained with hematoxylin and eosin and examined under light microscopy (BX41, Olympus, Japan) by two masked ophthalmic pathologists (MRK, SBH), in terms of integrity of the retinal layers and the presence of retinal hemorrhage, inflammation, and atrophy. Moreover, the immunohistochemical study for glial fibrillary acidic protein (GFAP) was also performed (polyclonal rabbit anti-glial fibrillary acidic protein, DAKO, Denmark) in the groups without any histopathological changes. Compared to the controls, increased GFAP immune reactivity in the retinal Muller cells in the treated groups was graded as remarkable or unremarkable. Histopathological changes indicating the retinal toxicity included: the absence of retinal layer integrity, presence of retinal hemorrhage, and/or inflammation, presence of retinal gliosis or atrophy, and the presence of remarkable GFAP immune reactivity.

## Phase II

Experimental CNV was induced in the right eye of 25 rats and the animals were randomized into the treatment group (14 animals) that received the maximum safe dose of IVI determined in phase I, and the control group (11 animals) that received intravitreal BSS. After 4 weeks, the animals underwent fluorescein angiography (FAG) and were then euthanized. The CNV areas were assessed by performing histopathological examinations.

### Laser-induced choroidal neovascularization

We used pigmented rats as a model for laser induced CNV, because rats’ retina is very similar to the human retina and it is homogenously pigmented and exhibits a high incidence of CNV after photocoagulation [22]. The rats were anesthetized and their pupils were dilated, as described above. By using a slit lamp infrared diode laser delivery system (Microlase, Keeler, Windsor, UK) and a noncontact 78 D lens, six laser spots (wavelength, 810 nm; spot size, 100 µm; duration, 0.1 s; and power, 150 mW) were applied between the major blood vessels in each eye. Development of a vapor bubble with each laser spot pointed to the rupture of the Bruch’s membrane.

### Fluorescein angiography

Four weeks after laser photocoagulation, fluorescein angiography (FAG) was performed to address any sign of CNV. Angiograms were taken under general anesthesia using a Heidelberg Retina Angiograph (HRA2, Heidelberg Engineering, Heidelberg, Germany) after intraperitoneal injection of 0.1 ml of 10 % fluorescein sodium (Fluocyne, Laboratories SERB, Paris, France). Pictures were interpreted by three masked retina specialists (HN, AR, RN). Leakage was characterized by the presence of a hyperfluorescent lesion on the late-phase angiograms and graded as: 0, no leakage; 1, slight leakage; 2, moderate leakage; and 3, prominent leakage [22, 23]. The leakage scores of the lesions in any rat were summed to yield a single value per rat per one observer. Then the scores were averaged between the three observers.

### Measurement of the choroidal neovascularization area in histopathological sections

The formalin-fixed eyes, as described above, were bisected axially and submitted for histological processing. After embedding into paraffin blocks, serial thin tissue sections were prepared and stained with hematoxylin and eosin. An attempt was made to serially section the entire CNV lesion. Sections were examined under light microscopy (BX41, Olympus, Tokyo, Japan) by two masked ocular pathologists (MRK, SBH). For each lesion, the section that was estimated to have the largest CNV was chosen and photographed using a digital camera (DP12, Olympus, Japan, Tokyo). Images were transferred to a computer and the area of CNV was delineated and calculated using the ImageJ software (ImageJ 1.48).

### Statistical analysis

All statistical analyses were performed by SPSS (Version 21.0, IBM Co., Chicago, IL, USA). The mixed model analysis was used to compare the results before and after injections in treated and control eyes. The Kruskal–Wallis test was used to compare the results in the groups at baseline. Comparison of the groups by adjusting the baseline values was performed by analysis of covariance (ANCOVA). Another mixed model was used to compare the leakage quantification, by considering the correlation of the results from the three observers. Also, we used regression analysis to obtain the trend of the dosage effect on the drug toxicity. In all the above analyses, the Bonferroni correction was considered for multiple comparisons.

*p*-values less than 0.05 were considered statistically significant.

## Results

### Phase I

One rat from each of the groups A, C, and F, two rats from the group D, and three from the group E did not survive, and therefore, were excluded from the study.

#### Electroretinography

Table 1 shows the mean of scotopic and photopic ERG amplitudes in the study groups at the baseline and 1 and 4 weeks after IVI. The ERG pattern and implicit times were almost identical in all groups. No significant differences in the photopic ERG results were observed between the groups and also in each group at the specified time-points of the study (*p* > 0.05). However, mean amplitude of b-wave in scotopic ERG was reduced significantly in groups A to D, 4 weeks after IVI (*p* < 0.05) and did not reveal a significant change in groups E and F (*p* > 0.05).

**Table 1:** Mean scotopic and photopic ERG a- and b-wave amplitudes in different groups at different time points

	Time		Group F	Group E	Group D	Group C	Group B	Group A	P value	
Photopic ERG a-wave amplitude (µv)	Base	Value	22.85 ± 28.57	19.48 ± 20.36	12.47 ± 5.91	12.45 ± 16.98	13.13 ± 9.19	12.86 ± 13.77	0.841^‡^	
	Week 1	Value	14.83 ± 11.72	11.17 ± 5.57	8.9 ± 6.59	10.84 ± 8.02	12.22 ± 7.73	10.38 ± 9.21	0.540*	
		Within P^†^	0.684	0.528	0.541	1.000	1.000	1.000		
	Week 4	Value	9.92 ± 4.46	8.25 ± 6.13	6.68 ± 5.73	10.46 ± 5.62	12.66 ± 10.67	8.07 ± 7.17	0.285*	
		Within P^†^	0.679	0.293	0.142	1.000	1.000	1.000		
Photopic ERG b-wave amplitude (µv)	Base	Value	39.55 ± 16.06	49.47 ± 16.29	36.25 ± 18.16	34.54 ± 30.04	47.82 ± 32.77	31.13 ± 15.47	0.721^‡^	
	Week 1	Value	28.83 ± 10.94	36.38 ± 17.26	28.88 ± 13.28	29.87 ± 17.7	44.72 ± 32.98	30.8 ± 17.31	0.277*	
		Within P^†^	0.165	0.523	0.222	1.000	1.000	1.000		
	Week 4	Value	35.48 ± 17.57	38.45 ± 31.76	26.73 ± 10.26	29.45 ± 12.36	25.18 ± 22.11	30.29 ± 25.66	0.853*	
		Within P^†^	0.934	1.000	0.205	1.000	0.276	1.000		
Scotopic ERG a-wave amplitude (µv)	Base	Value	77.19 ± 36.94	45.69 ± 24.72	34.43 ± 18.89	47.86 ± 36.06	51.19 ± 17.11	54.33 ± 17.06	0.079^‡^	
	Week 1	Value	68.69 ± 38.25	43.15 ± 42.86	31.21 ± 16.94	44.83 ± 24.19	43.18 ± 27.21	24.26 ± 14.82	0.024*	F Vs A
		Within P^†^	1.000	1.000	1.000	1.000	0.772	0.000		
	Week 4	Value	57.89 ± 11.08	54.52 ± 67.42	19.74 ± 15.09	30.86 ± 30.34	32.14 ± 24.89	25.64 ± 13.44	0.135*	
		Within P^†^	0.522	1.000	0.172	0.590	0.141	0.000		
Scotopic ERG b-wave amplitude (µv)	Base	Value	153.03 ± 83.46	186.2 ± 71.72	145.13 ± 45.81	126.6 ± 43.54	155.41 ± 52.43	154.32 ± 58.28	0.163^‡^	
	Week 1	Value	129.98 ± 48.91	169.2 ± 53.28	125.86 ± 87.19	73.5 ± 20.77	98.01 ± 39.99	70.66 ± 44.32	0.003*	F Vs C & A
		Within P^†^	1.000	1.000	0.928	0.026	0.061	0.000		
	Week 4	Value	102.13 ± 122.37	142.84 ± 68.11	88.57 ± 61.39	55.95 ± 29.69	90.23 ± 72.82	58.85 ± 41.12	0.195	
		Within P^†^	0.626	0.623	0.046	0.001	0.031	0.000		

^†^Based on Mixed analysis, adjusted for the multiple comparison by Bonferroni method

^‡^Based on Mann–Whitney test

* Adjusted for the baseline, based on analysis of covariance

#### Light microscopy

**Fig. 1:**
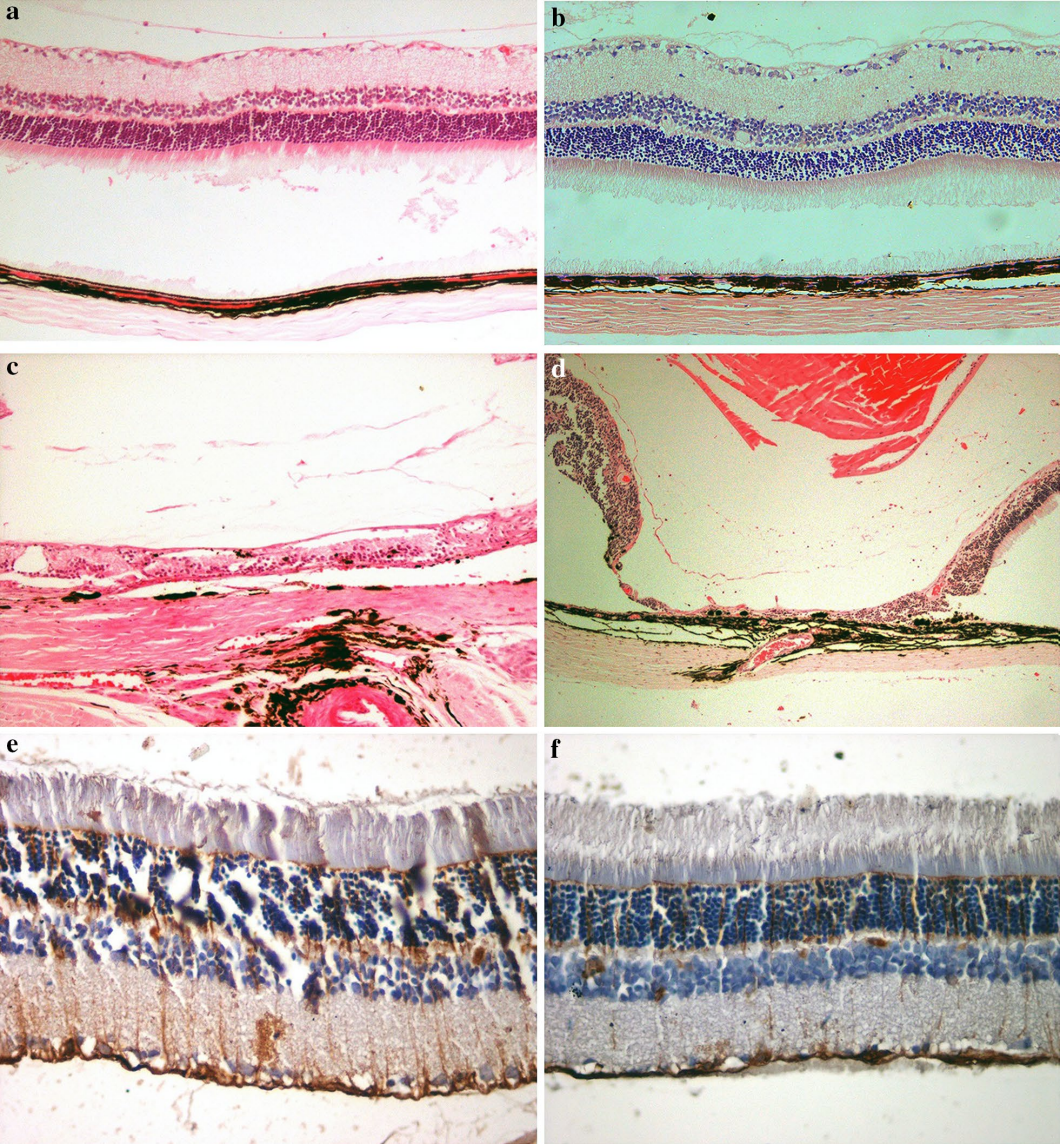
Histopathological and GFAP immune reactivity features in BSS and imatinib-injected eyes. Note the remarkable retinal layers in BSS-injected (**a**) and 40 µg imatinib-injected (**b**) eyes (hematoxylin and eosin, magnification ×200). There is severe retinal atrophy and disorganization together with pigment migration into the retina in 165 µg (**c**) and 330 µg (**d**) imatinib-injected eyes (hematoxylin and eosin, magnification ×200). GFAP immunoreactivity, as compared to a BSS-injected eye (**e**) was not significant in the 40 µg-injected (**f**) eye (magnification ×400). *GFAP* glial fibrillary acidic protein, *BSS* balanced salt solution

Histopathological analysis disclosed retinal toxic changes in the form of loss of retinal layer integrity and retinal atrophic changes in groups A to C. The histopathological features and GFAP immune-reactivity were unremarkable in groups D and E as compared to the controls (Table 2; Fig. 1). Given the ERG and histopathology results, 40 µg/5 µl was chosen as the maximum safe dose of IVI for phase II.

**Table 2:** Histopathological and GFAP immune reactivity findings in BSS and imatinib-injected eyes

	Group F (N = 9)	Group E (N = 7)	Group D (N = 8)	Group C (N = 9)	Group B (N = 10)	Group A (N = 9)
Intra and subretinal hemorrhage	0	0	0	0	0	0
Preserved retinal integrity	9	7	7	3	3	1
Preserved RPE integrity	9	7	7	3	4	2
Retinal atrophy	0	0	0	0	7	7
Remarkable GFAP	0	0	0	–	–	–

### Phase II

Out of 25 rats, three rats in the treatment and one in the control group expired before the final evaluation.

#### Choroidal neovascularization scores in fluorescein angiography

**Fig. 2:**
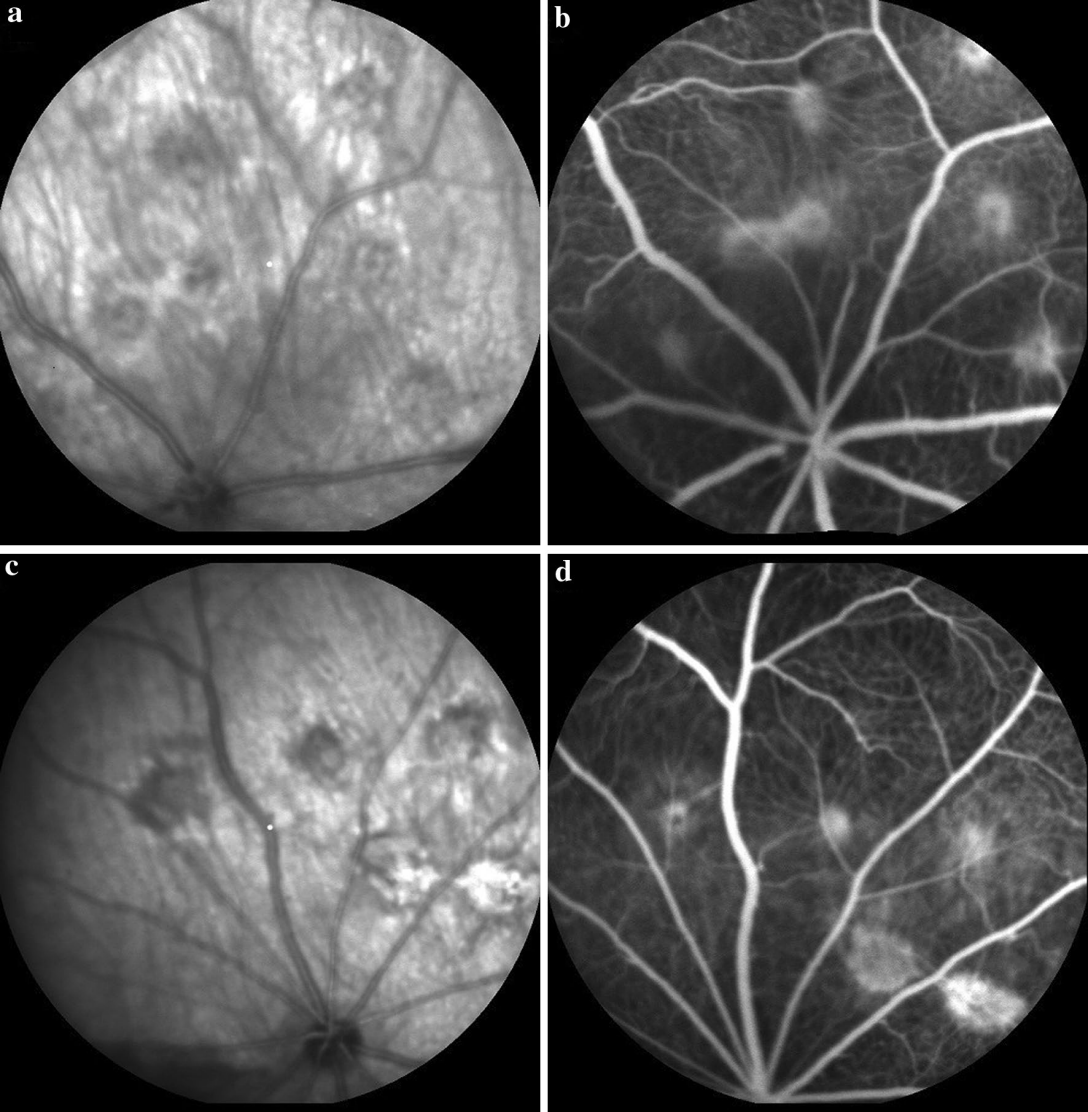
Fluorescein leakage from laser-burnt sites in treatment and control eyes. Infrared fundus photographs and fluorescein leakage from CNV lesions in the imatinib-treated (**a**, **b**) and control eyes (**c**, **d**), 4 weeks after laser application in the rat retina. Note the similar intensity of fluorescein leakage in both groups. *CNV* choroidal neovascularization

Four weeks after laser application, FAG revealed the development of CNV in the laser-burnt sites. The mean score of leakage in the imatinib-injected eyes was not significantly different from the controls (*p* = 0.619) (Fig. 2; Table 3).

**Table 3:** The fluorescein leakage score in the treatment and control eyes

	Control	Treatment	P value^§^
Number-leakage			
Mean ± SD	4.6 ± 2	4 ± 1.7	0.266
Median (range)	5 (1–6)	4 (2–6)	
Sum-leakage			
Mean ± SD	8.9 ± 6	9.8 ± 5.1	0.619
Median (range)	8 (1–18)	9.5 (3–18)	

^§^Based on Mixed model consider the correlation of the results for two observer

#### Choroidal neovascularization areas in histopathological sections

**Fig. 3:**
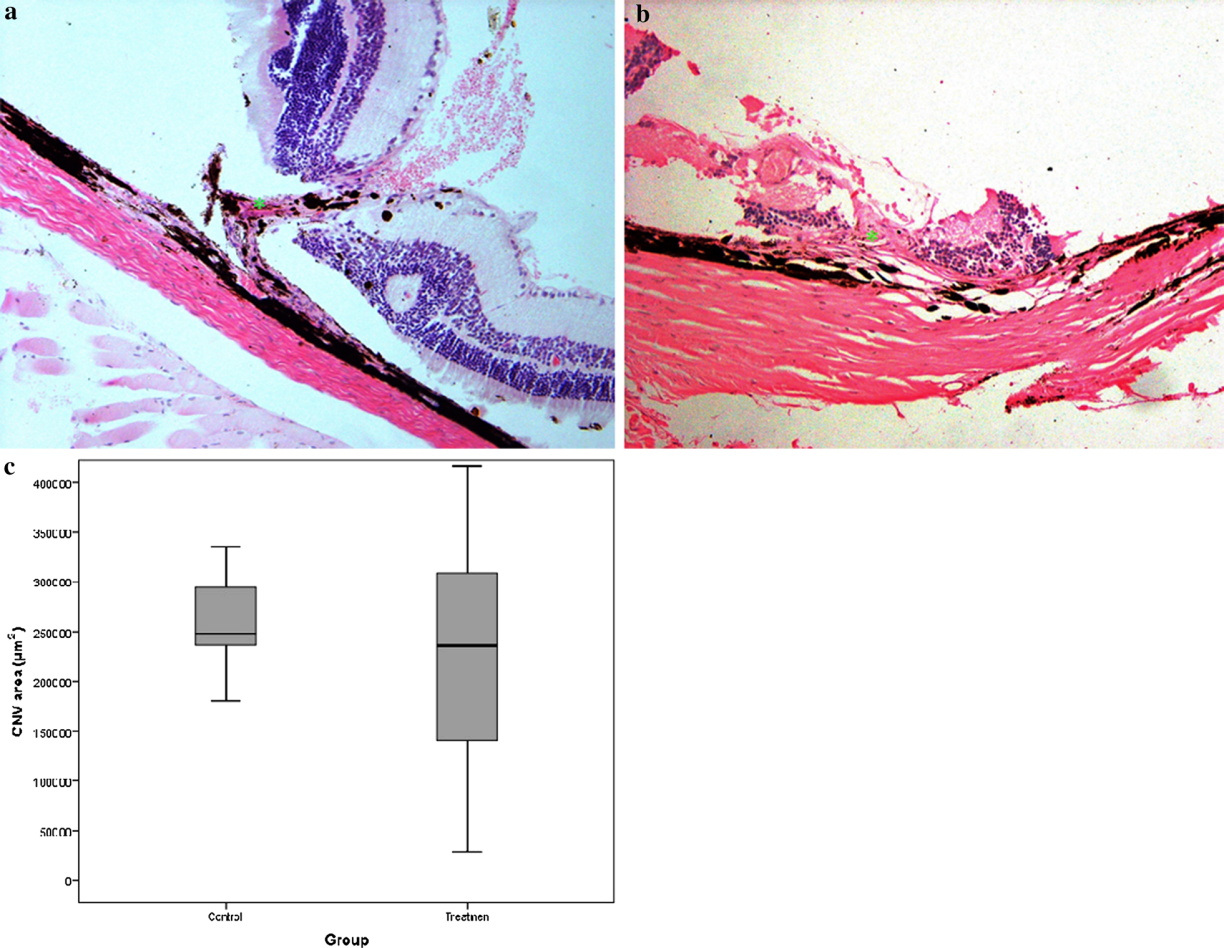
Choroidal neovascularization in histopathological sections and mean CNV area in treatment and control eyes. Note the presence of choroidal neovacular tissue (*green asterisk*) 4 weeks after 40 µg of IVI (**a**), with no significant difference from what is seen in a BSS-injected (**b**) eye (hematoxylin and eosin, magnification ×200). **c** The mean CNV area in the control and treatment groups was 258,836 µm^2^ ± 58,882 (median 247,946 µm^2^) and 277,401 µm^2^ ± 215,283 (median 236,262 µm^2^) respectively. The difference was not statistically significant (p = 0.499, based on the Mann–Whitney test). *IVI* intravitreal imatinib, *BSS* balanced salt solution, *CNV* choroidal neovascularization

The rats treated with IVI showed no significant difference in neovascular choroidal outgrowth compared to those in the controls; this finding was concordant with the FAG results. The average CNV area in the imatinib-treated eyes [mean (SE) 277401.1 (215282.9) µm^2^; n = 11 eyes] was comparable with the control eyes [mean (SE) 258835.7 (58881.6) µm^2^; n = 10 eyes] (*p* = 0.499) (Fig. 3).

## Discussion

In the present study, we found 40 µg of IVI as the maximum safe dose based on the ERG and histological findings. However, we failed to demonstrate any inhibitory effect on laser-induced CNV in an animal model, by using this dose of intravitreal imatinib. Also, the neovascular membrane size was variable in the treatment group, as we can observe with the box plot in Fig. 3. It might be related to the number of the rats in the treatment arm. Although we included 11 rats and it seemed enough for an animal study, this small sample size may be the reason for the scattered distribution noticed in the results (CNV areas). In addition, the final size of CNV area may be related to both the imatinib inhibitory effect and the extent of rupture in the Bruch’s membrane which could not be induced equally for all samples.

To the best of our knowledge, this was the first time that imatinib was used to inhibit laser-induced CNV lesions. Raimondi et al. recently found that imatinib inhibited physiological and pathological retinal angiogenesis in mice [24]. They revealed that intraperitoneal imatinib injection (100 mg/kg/day) for 5 days in an oxygen induced retinopathy (OIR) model of mice significantly reduced revascularization of avascular areas and effectively inhibited the formation of neovascular tufts by targeting neuropilin 1-dependent ABL1 activation in endothelial cells. They concluded that imatinib inhibited both physiological and pathological angiogenesis in the mouse retina in a VEGF- and VEGFR2-independent fashion via ABL1 inhibition. However, systemic usage of imatinib may induce both ocular and systemic side effects [25, 26]. Additionally, pathological neovascularization in OIR is different from that seen in CNV, in which the source of new vessels is choroidal vasculature. Some investigators studied the effects of other receptor tyrosine kinase inhibitors on laser-induced CNV in rats or mice. Park et al. investigated the effect of sorafenib, an orally administered, multi-targeted receptor tyrosine kinase inhibitor, on CNV progression in a rat CNV model, and showed that the drug inhibited laser-induced CNV [27]. Kang and co-workers evaluated the anti-angiogenic effects of the VEGF receptor tyrosine kinase inhibitor, cediranib, in an experimental laser-induced CNV model [28]. They found that oral administration of cediranib inhibited CNV growth. Pazopanib, a multi-targeted tyrosine kinase inhibitor was used by Takahashi and co-workers to inhibit laser-induced CNV development in rats [29]. They noticed that oral pazopanib inhibited CNV development. They also notified that treatment of established CNV with pazopanib, either systemically or by periocular injection, resulted in the regression of CNV. Kami and colleagues used SU5416 in mice, as a selective potent inhibitor of the receptor tyrosine kinase, intraperitoneally every other day for 14 days and showed that SU5416 suppressed fluorescein leakage from the photocoagulated lesion and inhibited laser-induced CNV formation partially [30]. On the other hand Honda and co-workers, in an experimental CNV model, used the liposomal form of the SU5416 and disclosed that only a single intravitreal injection of the drug reduces the CNV area significantly when compared with the control rats [31]. Unlike the above-mentioned studies, our survey did not show any advantages in using the intravitreal injection of the PDGF receptor inhibitor, imatinib, in a rat model of experimental CNV. This shows that PDGF only plays the role of a contributory factor in CNV formation and its inhibition per se cannot prevent CNV formation. Hence, VEGF inhibition seems to be the cornerstone of CNV inhibition. Furthermore, unlike sorafenib, cediranib, pazopanib, and SU5416, as multi-targeted tyrosine kinase inhibitors that prevent both PDGF and VEGF receptors, imatinib is a specific tyrosine kinase inhibitor that inhibits only the tyrosine kinase activity of PDGF receptors and does not have any influence on VEGF receptors.

In the phase I of the study, 40 µg/5 µl of IVI was determined as the maximum safe dose. Evaluating the intraocular toxicity of IVI in rabbits, Kitzmann et al. discovered that imatinib at a dose of 1.65 mg/0.1 ml caused extensive retinal histological toxicity in rabbits, while at lower doses of 825 µg/0.1 ml and 165 µg/0.1 ml, no sign of toxicity was observed [32]. Considering the vitreous volume in rabbit and rat eyes (1500 vs. 54 µl) we specified a greater safe dose of IVI compared to the Kitzmann report (0.74 vs. 0.55 µg/µl of vitreous volume). Furthermore, it should be noted that in the study by Kitzmann et al., only routine histological examinations were implemented to address the safe and toxic doses of IVI. They did not evaluate the GFAP immunoreactivity and electrophysiological tests, to detect subtle retinal toxicity that might have been induced by IVI. Moreover, in comparison with our study, they had used greater dose intervals. Dib and co-workers evaluated in vivo and in vitro toxicity of sunitinib malate, a multi-kinase inhibitor [33]. In the in vivo model, 0.1 cc of sunitinib was injected intravitreally in rabbits. No toxicity was observed with 12.5 mg/ml, but light microscopy showed that the 25 mg/ml solution damaged the photoreceptors layer. However, no functional changes in the electroretinogram were observed in any group. There was a disparity between functional and histopathologic results in 25 mg/ml concentration. Furthermore, five rabbits in each group might not be enough for toxicity evaluation.

Using a hand-made injectable form of imatinib was a shortcoming in our study. In addition, we injected the drug simultaneously when applying the laser injuries. Knowing that the half-lives of imatinib and its active metabolites are 18 and 40 h [34], and the fact that at least 2 weeks are needed for the laser-induced murine CNV to stabilize [35], one may conclude that the injected drugs in our study disappeared a long time before they had the chance of showing any effect. However, we supposed that the half-life and efficacy of imatinib in the vitreous, similar to many other drugs [36–39], would last long enough to influence the result. Nonetheless, further studies should be conducted to address the half-life of imatinib in the vitreous, as well as, the best time for the intravitreal injection of imatinib, to increase its inhibitory effect on laser-induced CNV. We injected BSS intravitreally in the control group, as we used a tiny amount of sulfuric acid in the imatinib preparation, using a neutral solution containing that minute amount of sulfuric acid in the control group could have been more acceptable.

## Conclusions

Intravitreal imatinib with the maximum safe dose of 40 µg/5 µl, which was determined in this study, had no inhibitory effect on laser-induced CNV in the rat model. However, imatinib might have a synergistic effect on the conventional anti-VEGF drugs in the treatment of CNV, which needs further investigations.

## Abbreviations

AMD: age-related macular degeneration
VEGF: vascular endothelial growth factor
CNV: choroidal neovascularization
PDGF: platelet-derived growth factor
IVI: intravitreal imatinib
BSS: balanced salt solution
ARVO: Association for Research in Vision and Ophthalmology
ERG: electroretinography
GFAP: glial fibrillary acidic protein
FAG: fluorescein angiography
